# How well does Malaysia achieve value for money in public sector purchasing of medicines? Evidence from medicines procurement prices from 2010 to 2014

**DOI:** 10.1186/s12913-020-05362-8

**Published:** 2020-06-05

**Authors:** NM Hamzah, P. N. Perera, R. P. Rannan-Eliya

**Affiliations:** 1 Pharmaceutical Services Division, Ministry of Health Malaysia, Lot 36, Jalan Universiti, 46200 Petaling Jaya, Selangor Malaysia; 2 Institute for Health Policy, 72, Park Street, Colombo 2, Sri Lanka

**Keywords:** Medicines price, Public procurement, Efficiency, Median price ratio, International reference price

## Abstract

**Background:**

Malaysia’s public healthcare sector provides a greater volume of medicines at lower overall cost compared to the private sector, indicating its importance in providing access to medicines for Malaysians. However, the Ministry of Health (MOH) has concerns about the continuous increase in the public sector medicines budget, and achieving efficiencies in medicines procurement is an important goal. The objectives of this study were to assess the overall trend in public sector pharmaceutical procurement efficiency from 2010 to 2014, and determine if the three different ways in which MOH procures medicines influence efficiency.

**Methods:**

We matched medicines from the public sector procurement report by medicine formulation to medicines with a Management Sciences for Health (MSH) International Reference Price (IRP) for each year. Price ratios were calculated, and utilizing the information on quantity and expenditure for each product, summary measures of procurement efficiency were reported as quantity- and expenditure-weighted average price ratios (WAPRs) for each year. Utilizing MOH procurement data to obtain information on procurement type, a multiple regression analysis, controlling for factors that can influence prices, assessed whether procured efficiency (relative to IRPs) differed by MOH procurement type.

**Results:**

Malaysia’s public sector purchased medicines at two to three times the IRP throughout the study period. However, procurement prices were relatively stable in terms of WAPRs each year (2.2 and 3.2 in 2010 to 1.9 and 2.9 in 2014 for quantity and expenditure WAPRs, respectively). Procurement efficiency did not vary between the three different methods of MOH procurement. Procurement efficiency of both imported originators and imported generics were significantly lower (*P* < 0.001 and *P* < 0.01) than local generic products, and medicine source and category influenced the procurement efficiency of each MOH procurement mechanism.

**Conclusion:**

The design of different medicines procurement mechanisms, along with the balance between ensuring competitive procurement prices and adhering to national industry and procurement policies, have not been able to achieve lower public sector medicines procurement prices (relative to IRP). Introducing pooled procurement options along with continuous monitoring of procurement efficiency and exploring ways to improve price competition among local and foreign suppliers is recommended.

## Background

Pharmaceuticals are a significant component of healthcare expenditure in Malaysia, accounting for 12.3–14.5%^1^ of total health spending from 2007 to 2016 [1, 2]. Reflecting Malaysia’s hybrid healthcare system, where public and private sectors operate in parallel on both the financing and delivery sides, the public and private sectors both play major roles in financing and providing medicines for Malaysians [3]. In 2016, the public sector financed approximately 32% of Malaysia’s total pharmaceutical expenditure of Malaysian Ringgit (MYR) 6.4 billion [1].

Public funds pay for the procurement and distribution of medicines through public facilities, with medicines usually provided free to patients at the point of service delivery. Public facilities consist primarily of facilities operated by the Ministry of Health (MOH), but these are supplemented by university hospitals that are managed by the Ministry of Education (MOE) and other services provided by other government agencies. The public sector accounted for more than two-thirds (70%) of total prescription medicines volume, measured in terms of standard medicine utilization units of defined daily doses (DDDs),^2^ but this supply accounted for only 48% of the total prescription medicines expenditure in 2014 [4]. The public sector supply of larger quantities at lower overall cost demonstrates the important contribution of this sector in providing access to medicines to Malaysians.

From 2007 to 2016, public sector medicines expenditure in Malaysia increased at an average annual rate of 9% to reach MYR 2.1 billion in 2016, which accounted for nearly 37% of public sector health expenditure [1]. This growth is an increasing concern for the MOH, whose budget finances the bulk of this. In the long term, achieving efficiencies in medicines procurement is an important requirement for sustaining the public healthcare system in Malaysia.

Malaysia’s public sector procures medicines using three mechanisms that are subject to public procurement regulations stipulated by the Ministry of Finance (MOF). These consist of: 1) a national concession agreement with one designated supplier; 2) national tenders; and 3) direct purchases by health facilities [5]. During the period examined in this study, the national concession agreement was with Pharmaniaga Logistics Sdn Bhd, a government-linked company (GLC) that took over the functions of the government central procurement service, including warehousing and distribution, in a form of privatization in the early 1990s. At present, public facilities can procure approximately 350 medicines, which are listed in the Approved Product Purchase List (APPL), directly from Pharmaniaga Logistics Sdn Bhd through the concession agreement at prices negotiated by MOH. Hereafter, purchases through the concession agreement are referred to as APPL. The second mechanism for medicines procurement involves MOH facilities ordering medicines via tenders managed centrally by the Procurement Division with technical support from the Pharmaceutical Services Programme. These centrally negotiated tenders on behalf of all public facilities are required for products where the annual purchase value exceeds MYR 500,000. For items, whose annual purchase value is between MYR 50,000 and MYR 500,000, facilities can directly purchase the items themselves, but must obtain a minimum number of quotations from suppliers registered with the government prior to procurement. For purchases less than MYR 50,000, the facilities are permitted to make direct purchases at their discretion. Regardless of the mechanism used, all purchases must be made from suppliers registered with the government and maintaining an operating presence in the country. In 2016, APPL, national tender and direct purchases accounted for 37, 43, and 20% of total MOH pharmaceutical spending [6].

Despite the central role that the public sector plays in financing and procurement of medicines, there is little published evidence on whether Malaysia’s public sector medicines procurement system achieves value for money. What limited information exists comes mostly from small-scale medicine price surveys [7–10] that have used the HAI-WHO pricing methodology [11]. Findings from these previous studies are subject to a number of limitations. First, their methodology does not formally incorporate information on medicine quantities when estimating the impact of price variations on aggregate price levels. Second, when such studies have reported that medicines are purchased at a higher or lower price than an international reference price (IRP), they have not been able to assess the overall impact on the medicines budget to place the findings in context, since they have not examined overall budget shares [11]. Third, these prior studies have considered only a relatively limited list of medicines (up to 50 medicines) often chosen without systematic sampling [11], which may not be reflective of the prices paid for the full range of medicines purchased by the public sector.

In our analysis, we incorporate information on purchase quantities to provide a more comprehensive assessment of the impact of procurement prices on the medicines budget and we assess all medicines, whose prices can be benchmarked against the Management Sciences for Health (MSH) IRP [12]. The objectives of our study were to assess the: 1) overall trends in public sector pharmaceutical procurement efficiency over a period of 5 years (2010–2014) by comparing Malaysia’s public sector procurement prices to MSH IRPs; and 2) whether procurement efficiency (measured as the price relative to IRPs) is affected by the procurement method used.

## Methods

### Data sources

#### IQVIA Malaysia pharmaceutical audit

We obtained data on public sector medicines prices for years 2010–2014 from the IQVIA Malaysia government purchase reports. IQVIA Malaysia tracks and reports medicines distributed through Malaysia’s public and private sectors through their Malaysian Government Purchase Report and Pharmaceutical Audit, respectively. IQVIA collects data from pharmaceutical distributors, and organizations responsible for administering purchases in the main medicine supply channels in Malaysia’s public sector – MOH hospitals and clinics, and three university hospitals. The collated data contain information on sales and quantity at the level of each product pack, along with information on active ingredients of the product, strength, dosage form, and pack sizes. The government purchase report also reports a weighted average price at the level of the product pack, and this is considered to be representative of the medicines procurement price paid by the public sector in Malaysia. Since this database tracks medicines channelled through both the MOH system as well as the university hospitals outside the MOH, and therefore captures the medicines channelled to the entire public health system, the IQVIA database was used for the main analysis.

#### MOH procurement database

The IQVIA data do not contain details of the procurement method used. To examine this, we obtained data on the method of procurement from the Pharmaceutical Services Division, MOH, which collects data on MOH procurements and compiles a single medicines procurement database. The database records cover a combination of APPL purchases for MOH facilities (hospitals, special medical institutions, health clinics and community clinics) provided directly by the appointed supplier, and both national tenders and direct purchases provided by individual facilities. The procurement data contains information on product name, active ingredients, strength, dosage form and pack size by the method of procurement (APPL, national tenders or direct purchases). Unlike the IQVIA data, the MOH procurement data do not include medicines procured by the Ministry of Education for university hospitals, so our analysis of the impact of procurement mechanisms on prices was restricted to medicines purchased only by the MOH, which represents 87% by value of total public sector expenditure.

#### MSH international drug price indicator database

We obtained IRPs for medicines from MSH International Medical Product Price Guide (www.mshpriceguide.org), which is produced in collaboration with the WHO and provides medicine reference prices categorized by supplier and buyer prices. Supplier prices represent the prices offered by for-profit and not-for-profit suppliers to developing country buyers for multi-source products. Buyer prices are actual prices paid by government agencies and development organizations for medicines purchased through international competitive bidding or tenders in developing countries, predominantly from Latin America, the Caribbean, and Africa. We carried out the main analysis using supplier prices because orders can be made on these prices unlike buyer prices that may only be available to certain buyers [13]. Multiple prices are reported for most medicine formulations from a number of sources and we used the median price (i.e., the median IRP) for price comparisons. In some cases, the median price is one single reported price. Prices reported in the price guide are obtained in the local currency are converted into U.S. dollars at the exchange rates fixed for each year [13].

### Analysis

#### Benchmarking prices against IRPs

We matched medicines from the IQVIA Malaysia’s government purchase report data by medicine formulation (active ingredient, strength, and dosage form) to medicines with an MSH IRP for the relevant year. During the period covered, IQVIA listed 1831 unique formulations as being purchased by the public sector. Table 1 summarizes the expenditure share, number of unique medicine formulations, and number of products associated with unique medicine formulations that we could match with IQVIA. These have been presented separately for all medicine formulations that had an IRP match each year, as well as for a subset of all items which were purchased and which had an MSH IRP in all five study years. We use this subset of medicine formulations as a consistent medicine price basket to make price trend comparisons across years. MSH predominantly covers WHO’s list of essential medicines and many non-essential medicines [13], combination medicines, and new and more specialized medicines are excluded. On average for the 5 years we studied, MSH supplier prices were available for approximately 725 products, which include different dosage forms and strengths of the same medicine.

**Table 1:** Summary of products matched to an MSH international reference price, 2010–2014

Year	All medicines			Medicine price basket		
	Medicine formulations	Products	Expenditure share (%)	Medicine formulations	Products	Expenditure share (%)
2010	254	624	25.9	205	532	24.8
2011	279	744	27.8	205	598	25.7
2012	267	725	28.9	205	604	27.2
2013	279	779	25.5	205	636	23.9
2014	266	751	28.2	205	628	25.7

Medicine formulation refers to a medicines active ingredient, strength, and dosage form that is used to match with medicines in the MSH price guide. A single medicine formulation can have a number of different products (branded and generic medicines) associated with it

The number of products is higher than the number of single medicine formulations since a single medicine formulation can be purchased as multiple different products (i.e.*,* originator product, branded generics, and generics) through the different purchasing mechanisms previously described. Furthermore, the same product can be available in two or more pack sizes, e.g., Ampicillin 125 mg/5 mL syrup (same brand) in two different pack sizes of 60 mL and 100 mL. The medicine price basket for which price comparisons with IRPs was possible in all years represents 24 to 26% of the total medicines budget during the 5 years of study. The basket also captures between 91 and 96% of medicines expenditures that can be compared to an IRP in any given year.

By value, the bulk (> 75%) of the medicine price basket consists of anti-infectives for systemic use, alimentary tract and metabolism medicines, and cardiovascular system medicines (Table 2). These three medicine categories along with dermatologicals and systemic hormonal preparations are relatively well represented in the basket, where the expenditure of medicines captured in the basket as a share of total expenditure exceeds more than 27% of each medicine category. However, for other medicine categories, the share ranged from 3% (antineoplastic and immunomodulating agents) to 19% (medicines used in the nervous system and respiratory system).

**Table 2:** Summary of composition and coverage of medicine price basket by medicine category (%), average from 2010 to 2014

WHO ATC level 1 category	Share of medicine formulations in basket (%)	Share of expenditure in basket (%)	Expenditure of medicines in basket as a percentage of total purchases by medicine category (%)
A – Alimentary tract and metabolism	15.1	39.6	58.9
B – Blood and blood forming organs	2.2	1.0	4.2
C – Cardiovascular system	16.0	17.7	27.4
D – Dermatalogicals	4.4	0.5	25.6
G – Genito-urinary system and sex hormones	2.0	0.5	3.5
H - Systemic hormonal preparations, excluding sex hormones and insulins	4.5	2.2	40.2
J – Antiinfectives for systemic use	27.1	21.5	29.1
L – Antineoplastic agents and immunomodulating agents	2.1	1.3	2.8
M – Musculo-skeletal system	3.7	1.2	10.9
N – Nervous system	11.2	8.9	19.1
P – Antiparasitic products, insecticides and repellents	0.9	0.2	8.2
R – Respiratory system	6.7	4.8	19.1
S – Sensory organs	4.0	0.7	12.1
V – Various			–
**Total**	**100.00**	**100.00**	**–**

We calculated the unit price of each matched item according to the price units used in the MSH price data. Some examples are price per tablet/capsule or per suppository, price per millilitre for oral liquids and price per ampoule/vial for some parenteral preparations. The Malaysian unit prices were then converted to USD using the official mid-year exchange rates for each year as reported by the World Bank World Development Indicators (WDI) [14]. For a given year, a price ratio for each product with an IRP match was calculated by dividing price per unit (in USD) by the median IRP per corresponding unit (in USD), with a 10% upward adjustment for the latter to incorporate shipping costs as recommended in the MSH price guide [13]. The calculation is shown as below:

$$ \mathrm{Price}\ \mathrm{Ratio}=\frac{\mathrm{Product}\ \mathrm{Price}}{\mathrm{Median}\ \mathrm{international}\ \mathrm{reference}\ \mathrm{price}\ast } $$

*If a median price is not available, the given single unit price is used.

Given that many price comparison surveys lack information on the relevant quantities and expenditures on medicine products, their usual approach is to calculate a median price ratio (MPR) by dividing the median price of all sampled products comprising a medicine formulation by the median IRP [12]. Since in our case, we had detailed data on the quantity and value of specific products within each formulation, we took into account the relative contribution by quantity and expenditure of products, to calculate weighted average price ratios (WAPRs) for each single medicine formulation with multiple products. We calculated two separate WAPRs: 1) a quantity-weighted average price ratio (QWAPR) that weights each product item price by the quantity of the product procured as measured by total DDDs; and 2) an expenditure-weighted average price ratio (EWAPR) that weights each product item price according to the monetary value of the product’s sales in a given year. Using these same methods, summary WAPRs were calculated for each year, to provide an overall assessment of public sector pharmaceutical procurement efficiency in a given year and to compare procurement efficiency (price relative to IRPs) across years.

#### Sensitivity analysis

We carried out a sensitivity analysis using MSH buyer prices as the price comparators. Unlike supplier prices, buyer prices can be specific to the government or regional procurement agency, or international organization that carried out the competitive bidding or tender [13]. Even though these prices may not be available for other governments and procurement agencies for purchase, they were considered to represent efficient bulk procurement. Comparisons were made with median buyer prices using the methods described above, while in an additional analysis we restricted the comparative price information available in the MSH data to the median buyer prices available from upper-middle income countries (UMICs) or predominantly UMIC country groups, to benchmark against comparable economic peers to Malaysia. The UMICs and UMIC groups included Botswana, Dominican Republic, Namibia, Peru, South Africa, Costa Rica (Costa Rica Social Security – CRSS), the System of Central American Integration (SICA), Barbados (Barbados Drug Service) and the Organisation for Eastern Caribbean States Pharmaceutical Procurement Service (OECS/PPS). Where there is group procurement for a number of countries, buyer prices from these agencies were included as comparators only if the countries in the group were predominantly UMICs.

#### Association between procurement method and MOH procurement efficiency

To assess the impact of procurement method on procurement prices, we matched the products in the medicines price basket to the MOH procurement data. From the IQVIA data we also obtained information on medicine source and category (local generic, imported generic and originator) and route of administration (eye preparations, inhaler, injectables, etc.). For the medicines selected, DDDs and expenditures were calculated from the MOH procurement data. To assess if procurement efficiency (product prices assessed relative to IRPs) varies by procurement method (national tenders, APPL and direct purchases) we carried out a multiple regression analysis by controlling for these variables. The regression estimated robust standard errors to take into account clustering as a result of medicine products (e.g., same brand) that occur across all 5 years of the study. Since the price ratios were not normally distributed, we used the log transformed price ratio as the dependent variable to minimize homoskedasticity. Significance of any association was assessed at alpha 0.05. Model diagnostics were carried out to ensure model assumptions were met.

We carried out all data management and analysis using Stata/SE 15 software [15].

## Results

For medicine formulations that had MSH IRP comparators, Malaysia’s public sector weighted average price ratio ranged between 1.9 to 3.5 times the IRP throughout the study period (Table 3). The trend in overall procurement prices was observed to be relatively stable over the years, except in 2014 where the QWAPR and EWAPR decreased by 14 and 9%, respectively, from 2010.

**Table 3:** Summary of price ratios for medicine price basket (*N* = 205), 2010–2014

	2010	2011	2012	2013	2014
Expenditure-weighted average price ratio (EWAPR)	3.2	3.5	3.5	3.3	2.9
Quantity-weighted average price ratio (QWAPR)	2.2	2.1	2.1	1.9	1.9
25th percentile price ratio^a^	1.0	0.9	1.0	0.9	0.8
50th percentile price ratio^a^	1.7	1.8	1.8	1.7	1.6
75th percentile price ratio^a^	3.8	4.1	3.9	3.9	3.9
Maximum price ratio^a^	122.4	128.1	94.6	150.2	69.4
Minimum price ratio^a^	0.1	0.1	0.1	0.1	< 0.1

^a^Unweighted price ratios relative to MSH international reference prices of all products included in the medicine price basket

**Fig. 1:**
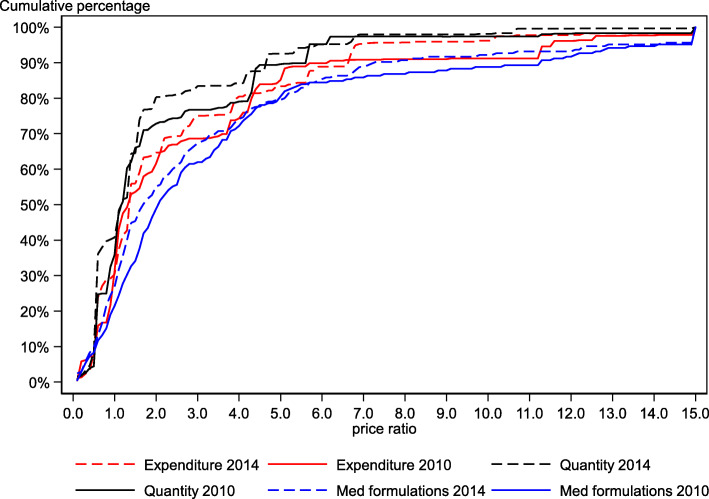
Cumulative percentage by price ratio for medicine price basket (*N* = 205), 2010 and 2014

This decrease in the WAPR in 2014 was a result of both a higher number of medicine formulations purchased at lower prices as well as lower priced medicines making up a larger proportion of the medicines purchased over the years (Fig. 1). The higher number of medicine formulations purchased at lower prices is observed by the shift in the cumulative frequency distribution for medicine formulations from right in 2010 to left in 2014. To further illustrate, the shares of medicine formulations procured at or below IRP, at or below twice the IRP, and at or below thrice the IRP were respectively 21, 49, and 62% in 2010, while it was 27, 55, and 67% in 2014. A similar leftward shift from 2010 to 2014 is observed for the cumulative quantity and cumulative expenditure curves. The total quantities of medicines (in DDDs) procured at or below IRP, twice the IRP, and thrice the IRP were respectively 36, 73, and 77% in 2010, and 41, 80, and 83% in 2014. In terms of expenditure, the total expenditure of medicines procured at or below IRP, twice the IRP, and thrice the IRP respectively, was 31, 62, and 69% in 2010 and 30, 65, and 75% in 2014 (Fig. 1). This shift in the quantity and expenditure curves indicates that a larger proportion of the total expenditure of the medicine price basket in 2014 is made up of medicines procured at lower prices than in 2010.

In 2014, approximately half of the 205 medicine formulations were purchased at or below twice the IRP: However, these medicines accounted for nearly 80% in terms of the total quantity and 65% in terms of expenditure. This difference in quantity and expenditures arises because a small volume of medicines accounts for a larger share of expenditures. This influences the overall EWAPR, increasing it by more than one point compared to the QWAPR (Table 3).

### Sensitivity analysis

The results of the sensitivity analysis using median buyer prices as well as median buyer prices of UMICs are presented in Table 4. The use of buyer prices from economically comparable countries does not change the main findings of the study – which are that Malaysia pays two to three times the MSH IRP, and that this showed no real change during the period of study. The overall price ratios obtained from the buyer price analysis was higher by about 3 to 22% than for supplier prices and may reflect country/regional-specific tender prices that generally may not be available to other buyers. This sensitivity analysis suggests that other UMICs obtain medicines at more competitive procurement prices than Malaysia.

**Table 4:** Summary of price ratios for medicine price basket, 2010–2014

	Quantity-weighted average price ratio			Expenditure-weighted average price ratio		
	Supplier (***N*** = 205)	Buyer (***N*** = 192)	Buyer-UMIC (***N*** = 164)	Supplier (***N*** = 205)	Buyer (***N*** = 192)	Buyer-UMIC (***N*** = 164)
2010	2.2	2.4	2.3	3.2	3.3	3.2
2011	2.1	2.3	2.5	3.5	4.3	4.6
2012	2.1	2.7	3.0	3.5	4.2	4.3
2013	1.9	2.3	2.7	3.3	3.9	3.8
2014	1.9	2.0	2.3	2.9	3.0	3.4

### Assessing procurement efficiency by MOH procurement method

To obtain information on procurement method, we matched each product in the medicines price basket (identified from IQVIA data) to the MOH procurement data by product name, dosage form and strength for each year. This matched 149 to 171 medicine formulations that accounted for 200 to 293 products in the MOH procurement data over the 5 years of study (Table 5). A complete match with products in the medicines price basket was not possible and is to some extent explained by products and strengths bought by non-MOH public sector facilities which are captured only in the IQVIA data.

**Table 5:** Summary of product matches of Ministry of Health procurement data with IQVIA medicines price basket, 2010–2014

Year	IQVIA medicines price basket		MOH procurement data	
	Medicine Formulations	Products	Medicine Formulations	Products
2010	205	532	149	200
2011	205	598	167	293
2012	205	604	167	262
2013	205	636	168	244
2014	205	628	171	266

IQVIA data includes medicines procured by MOH and non-MOH public healthcare institutions while MOH procurement data includes medicines procured by MOH healthcare facilities only

Of MOH procurement items that had matches to the medicines price basket, 47% were APPL items, followed by direct purchases (41%) and national tenders (12%), during all 5 years of the study (Table 6). The majority of APPL items were local generic products (70%) whereas half of the national tenders were originator products. On average, APPL purchase quantities are larger than quantities purchased through the other procurement mechanisms, which is to be expected given that items that are routinely needed in large volumes tend to be placed on the APPL list.

**Table 6:** Summary statistics of data used in models reported in Table 7, by procurement type, 2010–2014

Variables	Procurement Type		
	APPL (***n*** = 593)	National tender (***n*** = 150)	Direct Purchase (***n*** = 522)
Number of medicines formulations, (%)			
2010	14.5	19.3	16.3
2011	27.5	23.3	18.2
2012	19.1	22.0	22.2
2013	17.7	18.0	21.5
2014	21.2	17.3	21.8
Medicine source and category, (%)			
Local generic	70.2	32.0	36.4
Imported generic	20.2	18.0	30.3
Originator	9.6	50.0	33.3
Route of administration, (%)			
Injectables	30.0	30.7	15.5
Oral preparation	61.5	60.6	62.8
Other preparations	8.5	8.7	21.7
Median price ratio^a^ (Interquartile range)			
2010	1.58 (0.92–3.03)	2.08 (1.04–5.24)	2.77 (1.59–5.64)
2011	1.69 (0.98–5.89)	1.98 (0.97–5.89)	2.22 (1.41–5.00)
2012	1.61 (0.84–3.81)	1.24 (0.56–5.57)	2.16 (1.26–4.65)
2013	1.80 (0.83–3.84)	1.52 (0.94–6.12)	1.75 (1.14–4.08)
2014	1.50 (0.81–3.98)	1.35 (0.60–4.06)	1.69 (1.11–4.19)
Total quantity (‘000,000 DDDs), Mean (SD)	9.1 (33.5)	3.9 (16.7)	0.2 (0.6)
Total expenditure (‘000,000 MYR), Mean (SD)	2.0 (5.4)	1.6 (3.2)	0.1 (0.3)

^a^ Unweighted 50th percentile price ratio relative to MSH international reference prices of all products by procurement type for each year

We used regression analysis (Models 7A and 7B in Table 7) to assess the relationship between procurement efficiency as measured through medicines procurement prices relative to IRPs (i.e., price ratios) and other variables that might affect procurement prices. Both models control for route of administration, year of purchase, purchase volumes and expenditures. In addition, the second model (7B) also controls for medicine source and category. Model 7B indicates that local generics had lower price ratios compared to both imported generics (*P* < 0.01) and imported originators (*P* < 0.001).This is to be expected given that that there is more competition and lower prices available in the generics market, and given potential cost savings from local production of generics.

**Table 7:** Association between procurement efficiency and variables that affect procurement prices

	Model 7A		Model 7B	
	Coefficient (Standard error)		Coefficient (Standard error)	
Constant	0.793	(0.124)***	0.600	(0.128)***
Procurement type				
APPL (reference group)				
National tender	0.121	(0.213)	−0.164	(0.223)
Direct purchase	0.397	(0.141)**	0.180	(0.152)
Source and category				
Local generic (reference group)				
Imported originator			0.712	(0.205)***
Imported generic			0.422	(0.136)**
Route of administration				
Oral preparation (reference group)				
Injectable drugs	−0.370	(0.160)*	− 0.415	(0.158)**
Other preparations	−0.661	(0.199)***	−0.680	(0.210)***
Total quantity (‘000,000 DDDs)	−0.009	(0.003)**	−0.007	(0.002)**
Total expenditure (‘000,000 MYR)	0.036	(0.017)*	0.029	(0.015)
Year				
2010 (reference group)				
2011	−0.088	(0.066)	−0.044	(0.064)
2012	−0.116	(0.074)	−0.043	(0.070)
2013	−0.195	(0.083)*	−0.132	(0.080)
2014	−0.200	(0.083)*	−0.114	(0.082)
Adjusted R-squared	0.096		0.159	
Number of observations	1265		1265	

Dependent variable is the log of price ratio of MOH product procurement price relative to MSH IRP

* *P* < 0.05; ** *P* < 0.01; *** *P* < 0.001

In model 7A that does not control for medicine source and category, the relative price of items obtained through direct purchases were significantly higher than those obtained through the APPL (*P* < 0.01). However, this relationship did not hold in model 7B, which controls for medicine source and category, which suggests that the APPL and national tender mechanisms do not seem to achieve significant savings compared to direct purchases, with medicine source and category being a key variable that influences procurement efficiency.

The two models provide some evidence of economies of scale in purchasing with relative prices falling with increased quantities, but there was no consistent impact of increasing spending per item on relative prices. This could be because the relationship is complex, with prices for lower quantity items being higher and thus leading to higher overall expenditures. Larger volumes of medicines purchases were associated with significantly increased procurement efficiency (*P* < 0.01), while higher expenditures were associated with significantly lower procurement efficiency (*P* < 0.05). Comparison of price ratios of other study years with 2010 showed no significant variation in model 7B that controls for medicine source and category. Fixed price agreements that are negotiated for 2 to 3 years likely contribute to this relative stability of purchase prices relative to IRP.

Although there is some variation by medicine category, overall, originator products have higher price ratios followed by imported generics. Local generics are procured at the lowest relative prices (Table 8). By medicine category, originator products have higher median price ratios than generics except for alimentary tract and metabolism, blood and blood forming organs, dermatalogicals, systemic hormonal preparations (excluding sex hormones and insulins) and sensory organs medicines categories. Imported generics have higher median price ratios than local generics except for systemic hormonal preparations (excluding sex hormones and insulins) and respiratory system medicines categories.

**Table 8:** Price ratios by medicine source and category for all medicine products and by therapeutic category, (median (interquartile range)), 2010–2014

WHO ATC level 1 category	Local generics	Imported generics	Originator
All products	1.45 (0.94–2.57)	2.14 (1.07–4.34)	4.04 (1.50–6.88)
A – Alimentary tract and metabolism	1.44 (1.18–2.57)	2.09 (1.24–4.71)	1.52 (0.97–4.39)
B – Blood and blood forming organs	1.95 (1.59–16.78)	4.89 (2.80–8.19)	3.20 (2.25–3.27)
C – Cardiovascular system	1.42 (0.81–2.09)	3.09 (1.00–4.37)	7.43 (4.60–14.59)
D – Dermatalogicals	1.00 (0.44–1.50)	1.46 (1.12–1.70)	1.08 (0.36–1.80)
G – Genito-urinary system and sex hormones	0.42 (0.39–0.52)	1.16 (0.56–2.84)	4.59 (3.12–6.43)
H - Systemic hormonal preparations, excluding sex hormones and insulins	1.70 (1.06–2.13)	0.82 (0.58–1.33)	0.52 (0.23–0.91)
J – Anti-infectives for systemic use	1.80 (1.24–4.33)	2.74 (1.60–5.00)	4.45 (1.92–7.79)
L – Antineoplastic agents and immunomodulating agents	–	1.28 (0.46–2.55)	1.73 (1.70–2.13)
M – Musculo-skeletal system	1.11 (0.45–1.88)	4.28 (2.72–6.77)	5.13 (1.42–8.74)
N – Nervous system	1.17 (0.66–2.69)	2.24 (1.57–6.21)	4.35 (1.03–5.19)
P – Antiparasitic products, insecticides and repellents	1.79 (1.28–2.00)	3.14	–
R – Respiratory system	2.13 (0.62–3.57)	1.75 (1.06–4.25)	–
S – Sensory organs	1.13 (0.82–1.32)	2.36 (1.42–3.46)	0.65 (0.22–5.57)

## Discussion

Previous price comparison studies undertaken in Malaysia using the WHO/HAI pricing methodology have compared prices with MSH IRPs using such metrics as the median sample of prices of a medicine formulation, the lowest-price of available generics, and the price of the most-sold generic, and have considered only a small number of medicines in any single analysis [12]. In contrast, this study uses data for a more comprehensive range of medicines and incorporates information on the entire product range for a particular medicine formulation and their purchase quantities, to better understand the impact this mix of medicine products and their quantities can have on the medicines budget.

Overall, across all five study years (2010–2014), Malaysia purchased public sector medicines on average at two to three times the MSH IRPs. Overall, procurement efficiency as assessed relative to MSH IRPs was stable with a slight increase observed in 2014. This may suggest an improvement in procurement efficiency and can be assessed by extending this analysis beyond 2014. The improved efficiency in 2014 compared to 2010 are a combination of a larger number of medicines purchased at lower prices, and these medicines contributing to a larger share of the total quantity of the basket of medicines. These findings illustrate the importance of considering medicines quantities to understand the impact of pricing on the medicines budget.

In 2006, Babar et al. [7] surveyed 20 public hospitals in Malaysia, and for a small sample of 48 medicines found that the price ratio of 14 originator brands (IBs) were 2.41 times the IRPs, while for 26 most-sold generic equivalents (MSG) and lowest-priced generic (LPG) products, the price ratios were 1.56 and 1.09 times the IRP, respectively. The MOH Medicine Price Monitoring Survey from 2011 to 2014 at 45 public hospitals for 26 types of medicines found the MPR for the public sector to be 1.83 (2011), 2.19 (2012), 2.03 (2013) and 2.15 (2014) times higher than the IRP [9]. A recent survey in 18 public hospitals showed the MPR for 11 originator brand products was 1.2 and the MPR for lowest-priced generics was l.5 [10]. Our analysis, which finds somewhat similar or higher price ratios on average, is not directly comparable to these findings for several reasons. First, the price ratios in our study are presented as a weighted average (by either expenditure or quantity) and not simply the MPR. Second, our estimates are more comprehensive as they include all medicines that have a comparator price in MSH and are not restricted to a pre-defined basket of medicines. Third, the entire product range for a medicine formulation is included in the analysis and not just the median of the product range. We would argue that because of these differences in method, our results are a more meaningful and robust comparison of procurement price levels in Malaysia than earlier studies. Our findings also suggest that the WHO-HAI pricing methodology may under-estimate price ratios, at least in Malaysia.

Malaysia already implements several procurement practices that are known to facilitate obtaining competitive medicines prices. These include encouraging generic prescribing and substitution [16], price negotiation for APPL and national tender to obtain economies of scale, use of internal reference pricing (using prices in non MOH institutions and within therapeutic categories), and external reference pricing (with countries with similar economic status to Malaysia) [17]. The public sector also prioritizes the use of generic products, which makes up the majority of the medicines (approximately 76% are generic products with 64% by total value) as seen in our medicine price basket. In spite of this, Malaysia’s medicines prices on average are higher than MSH IRPs. We note that a set of indicators proposed by WHO to monitor access to essential medicines in the Western Pacific region has suggested that these nations should aim for an MPR of less than three [18] However, this recommendation is for a region that includes many island nations with small populations that struggle to obtain competitive prices for their medicines as they lack economies of scale and must pay higher transportation costs. In contrast, Malaysia which has good trade links, has greater purchasing power in the global market than many of the nations in the MSH database, and is in close proximity to major low-cost Asian producers, such as India and China, should be able to achieve substantially lower price ratios than it does. However, higher regulatory requirements in Malaysia such as compliance to Good Manufacturing Practice Standards set by Pharmaceutical Inspection Co-operation Scheme (PIC/s) members and conducting bioequivalence studies for some generic products could contribute to higher prices.

In Malaysia, national industry policies and government procurement policies give priority to local manufacturers and suppliers over international suppliers in competing for public procurement contracts [19]. This is in line with aspirations of the National Development Policy to stimulate the growth of local industries and accelerate economic growth whereby government procurement is used as a tool to achieve socio-economic and development objectives [5]. Malaysia has a local pharmaceutical industry consisting of 74 generic manufacturers that produce about 33% of the domestic market in terms of the total market value for pharmaceuticals [20]. The public sector supports the local industries by being the main buyer of the local generic products, with local generics making up 69% of the total quantity in DDDs for the medicine price basket in our study. When no local generic products are available, the remaining public sector procurement is sourced from imported generics (19% of total quantity in medicine price basket) and originator products (12% of total quantity in medicine price basket).

One possible factor that on average higher public sector procurement prices relative to IRPs is seen in Malaysia is likely due to fragmenting medicines procurement across public sector purchasers (MOH and university hospitals) and between three separate methods, as well as the design of each of these methods. Other associated reasons can be a lack of negotiating power since Malaysia also does not jointly procure with regional countries and allowing suppliers to simply charge higher prices in public sector purchasers. Although findings from the regression analysis that control for all variables indicate that procurement efficiency does not vary between the three different methods of procurement (relative to IRPs), medicine source and category was an important variable that influenced these findings. Products purchased through APPL are predominantly sourced from local generics (70%) compared to medicines sourced from local generics in national tenders and direct purchases (32 and 36%, respectively). Furthermore, our findings indicate that local generics are procured at lower relative prices compared to imported products. For APPL and national tenders, first choice of purchase are from local manufacturers and purchase of imported products are considered only if locally manufactured products are unavailable, which in some instances may result in higher prices being paid. Direct purchases can select the lower price between both local and imported products. In practice, as illustrated, this favours APPL purchases, where a lower proportion of APPL products are imported while there is a greater proportion of imported products purchased within both national tenders and direct purchases. Furthermore, purchase quantities are much larger for APPL products than quantities purchased from the other two methods. This is partly by design of the procurement method where the method of procurement is dependent on the total value of the medicines to be purchased.

Nevertheless, these differences that are related to both source and category of medicines between the three procurement methods likely contribute to the lower procurement efficiency of direct purchases compared to APPL observed when this variable is no longer controlled for in the regression analysis. This is likely due to a higher proportion of imported products and smaller quantities that may mean selection from within a few products or even single source products, and a lack of economies of scale in direct purchases by individual facilities compared to APPL. There was no significant price difference (relative to IRPs) between APPL and national tenders even though nearly 70% of national tenders are made up of imported products, perhaps due to greater economies of scale and confidential price discounts with national tenders enabling prices that are not different to prices of APPL products (relative to IRPs). However, APPL purchasers may translate into similar prices as national tenders due to strict performance criteria such as achieving delivery lags between 7 to 10 days compared to national tenders of usually 1 month and direct purchases between 1 to 3 months, respectively. Other associated costs include requirements to keep 3 months of stocks and door-to-door delivery that are not required in the other procurement mechanisms. Monitoring aggregate expenditures of direct purchases is important to assess if these aggregates exceed the threshold for national tenders (MYR 500,000), so that these medicines can be more effectively purchased at the central level (i.e., move direct purchases to central tender).

Although the relative procurement prices of local generics were lower than for imported generics, the large bulk purchase of local generic products was not effective in lowering prices towards parity with MSH IRPs. The current policies that prefer local generic medicines when there is import competition may lead to diminished pressure on local suppliers to reduce prices. Local producers that mainly cater for domestic consumption [21] may also enjoy lower economies of scale and so may not be able to meet MSH IRPs. A paper by Azmi IM et al. (2001) highlighted that the local industry does not have any cost advantages stemming from cheap labour or resource abundance, and that small-scale production with limited research and development capacity [22] may be possible reasons that prices are not competitive as compared to IRPs. Local generics are mostly purchased via APPL with price revision every 3 years. It is recommended that price-volume adjustments of APPL products should be negotiated more frequently. One option to ensure greater price competition would be to introduce a fixed price handicap to local producers in the procurement process instead of arbitrary preferences to select local suppliers. Other options include encouraging early entry of generics by increasing the capability of local generic production, and providing incentives for local producers to penetrate the export market such as through offtake agreements and other incentives designed to promote price competition in the procurement process.

There should be price transparency at all levels of the supply chain and the government should work towards sharing price information with countries in the Asia-Pacific region where initial steps are already taking place with the development of The Price Information Exchange for Essential Medicines” (PIEMEDS) web-based system (WHO WPRO, WHO SEARO) [23]. This is in line with the successful resolution laid out on ‘Improving the Transparency of Markets for Medicines, Vaccines, and Other Health Products’ at the World Health Organization 72nd World Health Assembly (WHA) [24]. A national reimbursement system would allow higher purchasing power to negotiate prices for the whole Malaysian market, and currently pooled procurement for public sector (including the Ministries of Health, Education and Defence) is underway for selected medicines. Initial work assessing the feasibility of pool procurement within ASEAN region is also being undertaken. Improvements such as centralized quotations at state level are also being implemented to ensure health facilities within a state receive a fixed negotiated price.

Several limitations in this study are noted. Although we included all products that had an MSH IRP comparator, in our analysis this only still represents 24–26% of the total value of the public sector budget. The soundness of the median price as a comparator depends on the number of prices used to determine the median IRP for each medicine. When only a few prices are available or when there is only one price available as the comparator, the price ratios for specific medicines can be skewed due to a particularly high or low IRP, although this will have minimal effect on aggregate comparisons made for large numbers of products. In addition, packaging could affect the unit prices, but this was not considered in the analysis due to unavailability of this information in the MSH medical products price guide. Further investigation is necessary as differences in quality of products and patent status are not accounted for in this analysis. Finally, while we have used the widely used MSH IRP for our analysis, the MSH database has limited geographical coverage, particularly in the Asia-Pacific region. Future studies that include data points from regional countries are recommended as they may better reflect regional patterns in pharmaceutical procurement.

## Conclusions

Malaysia’s public sector purchases medicines on average at prices two to three times the IRP, which suggests considerable room for price savings. There was little change in this disparity during 2010–2014, except for a small reduction in 2014. Medicine source and category is a key variable that influences procurement efficiency. Having controlled for source of medicines, the most important procurement mechanisms, the concession agreement and centrally managed tenders, does not appear to achieve substantially lower prices than direct purchasing by facilities. However, the large differences in composition of the three procurement methods by medicine source (local vs imported) and category (generic vs originator) (e.g., more imported originators in national tenders) make comparisons of procurement efficiency between the three procurement methods limited.

Even though the MOH carries out a number of policies considered to be effective methods for negotiating lower prices, the public sector pharmaceutical procurement has to adhere to procurement mechanisms as stipulated in the public procurement procedures. As such, there are challenges in striking a balance between ensuring competitive public sector medicines procurement prices and adhering to public procurement procedures. It is recommended that Malaysia identify the key medicines and medicines categories that have the largest impact on the medicines budget to explore the possibility of targeting aggressive price negotiations and other innovative ways of procuring medicines in these categories. Other forms of incentives should be introduced to allow local industry to offer competitive prices in public procurement.

## Data Availability

The public sector procurement data that support the findings of this study are available from IQVIA (formerly known as QuintilesIMS) Malaysia. Restrictions apply to the availability of these data, which were used under license for the current study, and are not publicly available. Data are however available from the authors upon reasonable request and with permission of IQVIA Malaysia. Ministry of Health procurement data are available from the corresponding author upon reasonable request and international reference price data is available in the public domain at www.mshpriceguide.org.

## Abbreviations

APPL: Approved Product Purchase List
DDD: Defined daily dose
EWAPR: Expenditure-weighted average price ratio
HAI: Health Action International
IRP: International reference price
MOF: Ministry of Finance
MOH: Ministry of Health
MPR: Median price ratio
MSH: Management Sciences for Health
MYR: Malaysian Ringgit
QWAPR: Quantity-weighted average price ratio
UMIC: Upper Middle-Income Country
WAPR: Weighted average price ratio
WHO: World Health Organization

## Footnotes

1 The total pharmaceutical expenditure is derived from the sales data captured at the wholesale level.

2 The DDD is the assumed average maintenance dose per day for a drug used for its main indication in adults.
